# A systematic review of the current challenges of bedside teaching for medical students in the UK

**DOI:** 10.1186/s12909-026-09931-z

**Published:** 2026-07-15

**Authors:** Ikechukwu Roland Chukwudi, Kelvin Kanayo Nwabueze, Anuoluwapo Olanrewaju

**Affiliations:** 1 Acute Medicine Department, Liverpool University Hospitals’ NHS Foundation Trust, Liverpool, L9 7AL UK; 2 Acute Medicine Department, Watford General Hospital, West Hertfordshiire NHS Trust, Watford, WD18 0HB UK; 3 Department of Medicine, University College Hospital, Ibadan, Nigeria

**Keywords:** Bedside teaching, Medical education, United Kingdom, Qualitative systematic review, NHS

## Abstract

**Purpose:**

Bedside teaching (BST) has declined in the UK despite longstanding educational interventions. We sought to outline current barriers and synthesise experiences of medical students and clinical educators to understand organisational dynamics constraining BST.

**Method:**

We conducted a qualitative systematic review following PRISMA 2020, registered in PROSPERO (CRD420251033043). MEDLINE, Embase, CINAHL, and Cochrane Library were searched for 2014 to April 2025. We included UK qualitative or mixed-methods studies on barriers to BST; non-UK studies, reviews, and editorials were excluded. Two reviewers independently screened and appraised studies using the Critical Appraisal Skills Programme (CASP) checklist. Thematic synthesis was performed with line-by-line coding and iterative team discussion.

**Results:**

Four interconnected themes emerged: (1) institutional marginalisation of undergraduate teaching (service pressures, limited recognition/resources), (2) educator constraints and professional isolation (limited training/feedback, competing demands), (3) learner unpreparedness and engagement challenges (insufficient exposure and confidence), and (4) patient-related ethical and practical barriers (reluctance, privacy, infection concerns). These themes formed a self-reinforcing organisational cascade that sustains BST decline.

**Conclusions:**

Barriers to BST in the UK are systemic rather than isolated, reflecting structural misalignment between service demands and educational priorities. Sustainable improvement requires organisational reform and resourcing that explicitly prioritise bedside teaching within clinical practice, alongside continued investment in the educators who deliver it.

**Supplementary Information:**

The online version contains supplementary material available at https://doi.org/10.1186/s12909-026-09931-z.

## Introduction

Bedside teaching (BST) has traditionally been central to medical education. Beyond technical skills, it is the primary locus for learning professional behaviour, patient interaction, and clinical reasoning. However, bedside teaching in the UK has declined substantially over recent decades, with current students receiving significantly less bedside instruction than previous generations [1, 2].

The literature has identified numerous contributors to this decline, including workload pressures, lack of educator training, and patient reluctance [3, 4]. While valuable, these studies typically frame barriers as discrete problems requiring individual solutions, such as teaching awards or protected time. Yet, despite decades of such interventions, the decline persists. This persistence suggests that the individual barriers often cited, anxious students or busy doctors, may be symptoms of deeper organisational dynamics rather than root causes.

The UK National Health Service provides a critical context for investigating this phenomenon. Recent reforms have intensified efficiency targets. The NHS Long Term Workforce Plan commits to doubling medical student numbers to 15,000 by 2031 without a proportionate expansion of teaching infrastructure [5, 6]. This creates a tension where healthcare organisations designed for patient throughput are increasingly asked to deliver education without the necessary structural adaptation.

We conducted this systematic review to outline current barriers to bedside teaching, using “barriers” broadly to include institutional, educator-level, learner-level and patient-related factors that impede the delivery, quality or sustainability of bedside teaching. We also sought to synthesise the experiences of medical students and clinical educators, including allied health professionals, resident doctors and consultants, to understand the organisational dynamics that constrain bedside teaching in the UK.

## Methods

### Design and search strategy

We conducted a systematic review of qualitative evidence following PRISMA 2020 guidelines [7], with the protocol registered in PROSPERO (CRD420251033043). We searched MEDLINE, Embase, CINAHL, and the Cochrane Library in April 2025 for studies published between January 2014 and April 2025. Search terms combined controlled vocabulary and keywords related to bedside teaching, barriers, medical students, and the UK context. We restricted inclusion to qualitative or mixed-methods studies investigating barriers to bedside teaching in UK clinical settings, excluding non-UK studies, reviews, and editorials (see Table S1 for the full search strategy).

### Study selection and appraisal

**Fig. 1:**
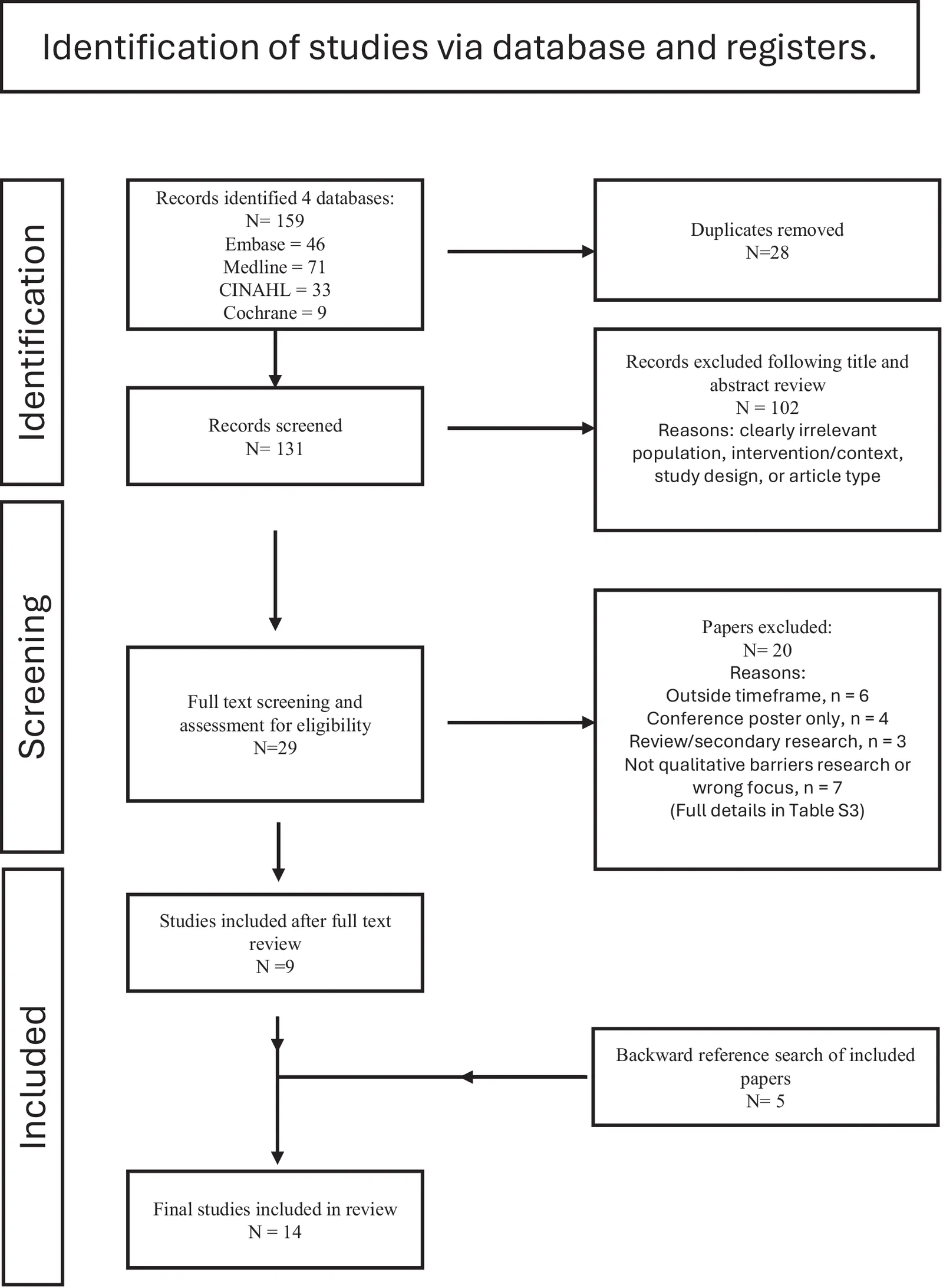
PRISMA 2020 flow diagram of study identification, screening, eligibility, and inclusion

Two reviewers independently screened titles and abstracts, followed by full-text review. Disagreements were resolved through discussion or consultation with a third reviewer. Twenty-nine full-text articles were assessed and nine studies met all inclusion criteria (see Fig. 1 and Table S3). We appraised study quality using the Critical Appraisal Skills Programme (CASP) qualitative checklist (see Table S2). Following initial calibration to ensure consistent application of criteria regarding reflexivity and theoretical frameworks, we reached consensus on all ratings. The included studies generally demonstrated strong methodological quality.

A reverse-reference search of the included studies identified a further five eligible papers, bringing the final total to 14 studies included in the review. Considering that a significant proportion of medical student rotations occur in primary care, studies referring to teaching in primary care settings outside the hospital environment, but still involving direct patient contact, were considered a form of bedside teaching.

### Data synthesis

We employed thematic synthesis to analyse findings. Two reviewers independently coded data line-by-line to develop descriptive themes. Through constant comparison and iterative team discussions, we refined these into analytical themes that transcended individual studies to identify patterns across the dataset. NVivo 15 software was used to manage the analysis.

### Reflexivity

The author team comprises three clinicians actively engaged in bedside teaching for medical students, two of whom currently practise in the NHS with a combined nine years of UK clinical experience. All three authors completed their undergraduate training outside the UK (University of Ibadan, Nigeria), within a system that emphasises hands-on, experiential learning at the bedside. This positionality shaped our engagement with the data in two ways. Our training background may have sensitised us to the value of practical instruction and predisposed us towards interpreting its decline as a loss, while our position as clinicians trained outside the UK but working within it offered an outsider-insider perspective, allowing us to question assumptions about how bedside teaching is organised and valued that may appear self-evident to those trained within the system. To maintain rigour, we held regular reflexive discussions throughout screening, coding and synthesis, in which interpretations were challenged and alternative explanations were considered, particularly the possibility that the barriers we characterised as systemic could instead reflect individual or pedagogical factors.

## Results

### Study characteristics

We included 14 qualitative studies published between 2014 and 2025. Studies were conducted across hospital, community and general practice settings involving medical students only (*n* = 2), educators only (*n* = 9), both medical students and clinical educators (*n* = 2), and patients only (*n* = 1). Sample sizes ranged from 5 to 368 participants. Study designs comprised semi-structured interviews (*n* = 3), focus groups (*n* = 3), and questionnaire surveys (*n* = 5), with three studies employing multiple methods (*n* = 3). See Table 1 for detailed study characteristics.

**Table 1:** Characteristics of included studies

Author (Year)	Title	Aim	Population	Design/Data Collection	Sample size (n)	Analysis Method	CASP Appraisal
Gadoud et al. (2018)[19]	A pilot study of interprofessional palliative care education of medical students in the UK and USA	Explore non-medical palliative care health professionals’ (NMPCHP) perceptions of interprofessional training of medical students	Clinical educators (non-medical palliative care professionals)	Focus groups (three UK sites)	13	Constant comparative analysis	High
Zamoyski & Alberti (2018) [8]	An evaluation of the views of GP specialty trainees on their involvement in medical student teaching	Assess GP specialty trainees’ perceptions of teaching medical students for their development as future GPs and educators	GP specialty trainees (Clinical educators)	Semi-structured interviews	5	Thematic analysis	High
Harrison et al. (2019) [9]	Barriers to involving GP specialty trainees in the teaching of medical students in primary care: the GP trainer perspective	Explore GP trainers’ perspectives on trainees’ teaching roles	GP trainers (Clinical educators)	Focus groups	31	Thematic analysis	High
Harris et al. (2024)[24]	Experiences as a clinical teaching fellow: interviews with clinical teaching fellows in the West Midlands	Explore clinical teaching fellows’ experiences at the end of their year in post	Clinical teaching fellows (Clinical educators)	Semi-structured interviews	9	Thematic analysis	High
Barber et al. (2019) [10]	Facilitators and barriers to teaching undergraduate medical students in general practice	Identify facilitators and barriers to GP involvement in undergraduate teaching to inform tutor recruitment	GPs (clinical educators)	Semi-structured interviews	24	Thematic analysis	High
Jones et al. (2017) [11]	GP registrars teaching medical students: an untapped resource?	Evaluate GP training posts’ role in undergraduate medical education	GP registrars (Clinical educators) and medical students	Semi-structured interviews and focus groups	26	Thematic analysis	High
Gray et al. (2017) [18]	Medical students’ perceptions of bedside teaching	Describe medical students’ perceptions of bedside teaching by junior doctors versus consultants	Medical students	Online questionnaire (open-ended responses)	46	Descriptive statistics and thematic analysis	High
Shone et al. (2025) [12]	Patient perspectives on bedside teaching during a pandemic	Evaluate patients’ views on participation in bedside teaching during COVID-19	Patients	Open-ended questionnaire	50	Descriptive statistics and thematic analysis	High
Harding et al. (2015) [13]	Provision of medical student teaching in UK general practices: a cross-sectional questionnaire study	Quantify medical student exposure to general practice teaching and compare with postgraduate provision	GP educators (Clinical educators)	Online questionnaire (open-ended responses)	29	Descriptive statistics and thematic analysis	High
Blake et al. (2014) [14]	Teaching musculoskeletal examination skills to UK medical students: a comparative survey of Rheumatology and Orthopaedic education practice	Assess undergraduate MSK teaching methods, barriers, and attitudes toward the GALS screening tool	Rheumatology and Orthopaedic trainees and consultants (Clinical educators)	Open-ended questionnaire	76	Descriptive statistics and thematic analysis	High
Jones & Rai (2015) [1]	The status of bedside teaching in the United Kingdom: the student perspective	Describe medical students’ national perspectives on bedside teaching	Medical students	Online questionnaire (open-ended responses)	368	Descriptive statistics and thematic analysis	High
Chew & O’Dwyer (2020) [15]	Undergraduate medical education: a national survey of consultant radiologists	Assess radiologists’ role in undergraduate medical education in Scotland	Consultant radiologists (Clinical educators)	Online survey with open-ended items	102	Descriptive statistics and thematic analysis	High
Al-bedaery et al. (2022) [16]	Undergraduate medical teaching with remote consultations in general practice: a realist evaluation	Understand facilitators and barriers to teaching via remote consultations and perceived educational impact	Medical students and GP tutors (Clinical educators)	Open-ended questionnaire and semi-structured interviews	68 (49 students, 19 tutors)	Thematic analysis	High
Hayden et al. (2021) [17]	Understanding junior doctors’ experiences of teaching on the acute take: a qualitative study	Explore junior doctors’ experiences of supporting undergraduate education on the acute take	Junior doctors (Clinical educators)	Focus groups	14	Thematic analysis	High

*Abbreviations* *GP * general practice, *GPST * GP specialty trainee, *NMPCHP * non‑medical palliative care health professional, *MSK * musculoskeletal, *GALS * gait–arms–legs–spine, *CASP * Critical Appraisal Skills Programme (qualitative checklist)

### Overview of thematic analysis

Thematic analysis identified four interconnected domains describing barriers to bedside teaching in UK medical education (Table 2). These themes reflect institutional, educator, learner, and patient-level factors that contribute to the decline in bedside teaching opportunities. The themes are presented with their constituent subthemes below.

**Table 2:** Themes mapped to subthemes and illustrative quotes

Major Theme	Subthemes	Illustrative Quotes
1. Institutional Marginalisation of Undergraduate Teaching	1.1 Service Efficiency Over Educational Activity	"Teaching is routinely sidelined by clinical workload… a low priority relative to waiting list management."
	1.2 Absence of Formal Recognition and Resource Allocation	"Lack of job plan allocation and active recruitment into teaching roles… teaching remains voluntary."
	1.3 Physical Infrastructure Deficits	"Lack of physical space for student teaching… [leading to] student perception of missing valued hospital-based teaching opportunities."
	1.4 Disconnection Between Academic and Clinical Structures	"Weak links with medical school… teaching not aligned with clinical practice leaves students confused."
2. Educator Constraints and Professional Isolation	2.1 The Anxiety of the Amateur Educator	"Teacher anxiety about teaching… [compounded by] limited teacher training."
	2.2 Professional Isolation and Lack of Feedback	"Minimal feedback to interprofessional educators… lack of recognition or voice in teaching decisions."
	2.3 Competing Clinical and Educational Demands	"Struggle to balance teaching with clinical practice… often forces clinicians to work beyond contracted hours."
	2.4 Inconsistent Teaching Quality	"Variability in quality of teaching… consultant intimidating teaching style… or teaching is simply left to chance."
3. Learner Unpreparedness and Engagement Challenges	3.1 Insufficient Exposure and Confidence Deficits	"Low learner confidence limits bedside skill development… anxiety about making mistakes in front of peers."
	3.2 Professionalism and Disengagement	"Student professionalism challenges… reduced attention and engagement when not directly involved."
	3.3 Impact of Remote Consultation Modalities	"Remote consultation format limits depth of reflection and learning… reduced face-to-face patient-doctor interaction."
4. The Patient-Related Ethical and Practical Interface	4.1 The Burden on the Patient	"Patient reluctance to participate… perceived burden on patients."
	4.2 Infection Risk Concerns	"Concern about COVID transmission… high-risk groups limit teaching."
	4.3 Physical Environment and Privacy	"Lack of privacy in teaching… student embarrassment… discomfort with physical proximity."

### Theme 1: Institutional Marginalisation of Undergraduate Teaching

Across settings, bedside teaching was structurally deprioritised relative to clinical service delivery and research activities. This marginalisation operated through resource allocation, physical infrastructure, and organisational culture.

#### Subtheme 1.1: Service Efficiency Over Educational Activity

At the institutional and organisational level, teaching was consistently described as being “sidelined by clinical workload”. Participants reported that teaching capacity was often the first activity to be lost when clinical pressures mounted, with teaching viewed as “low priority” relative to waiting list management. “Excessive clinical workload and staffing shortages” were cited as primary barriers, and teaching frequently relied on staff “goodwill” rather than protected time [9, 10, 14, 15, 17].

#### Subtheme 1.2: Absence of Formal Recognition and Resource Allocation

Teaching was frequently absent from formal employment structures. Studies identified “lack of job plan allocation and active recruitment into teaching roles”. “Limited resources and funding for teaching” prevented departments from backfilling clinical time when staff engaged in education [9, 13, 15, 17].

#### Subtheme 1.3: Physical Infrastructure Deficits

Hospital environments optimised for bed capacity had eroded dedicated educational spaces. Multiple studies reported a “lack of physical space for student teaching”. The absence of seminar rooms or private ward space prevented confidential briefing and debriefing, leading to “student perception of missing valued hospital-based teaching opportunities” [9–11, 17].

#### Subtheme 1.4: Disconnection Between Academic and Clinical Structures

Clinicians reported feeling disconnected from medical school curricula. This manifested as “weak links with medical school” and “unclear learning objectives”. In some specialties, “teaching not aligned with clinical practice” left students uncertain about the relevance of their clinical exposures [9, 10, 14, 18].

### Theme 2: Educator Constraints and Professional Isolation

The institutional environment created specific pressures for individual clinicians responsible for teaching, characterised by inadequate preparation, limited feedback, and competing demands.

#### Subtheme 2.1: The Anxiety of the Amateur Educator

Significant “teacher anxiety about teaching” was reported, particularly among junior clinicians. Many felt exposed due to “limited teacher training”. General Practice (GP) trainees described “increased stress during exam periods” and “discomfort teaching primary care-specific topics”, reflecting “limited depth of knowledge compared to senior clinicians” [8, 9, 12, 17].

#### Subtheme 2.2: Professional Isolation and Lack of Feedback

Educators frequently worked without structured support. Studies highlighted a “lack of structured feedback systems” and “minimal feedback to interprofessional educators”. Educators also reported a “lack of recognition or voice in teaching decisions”, with teaching staff “not always involved in curriculum design” [9, 10, 15, 19, 24].

#### Subtheme 2.3: Competing Clinical and Educational Demands

Individual clinicians described the daily reality of trying to deliver teaching within these constraints. Educators reported a “struggle to balance teaching with clinical practice” and often needed to “work beyond contracted hours to meet teaching demands”. Some expressed a preference for teaching postgraduate trainees over undergraduates, as postgraduates could contribute more directly to service delivery while learning [9, 10, 24].

#### Subtheme 2.4: Inconsistent Teaching Quality

The absence of standardisation led to “variability in quality of teaching across specialties”. Negative experiences included “consultant intimidating teaching style” or teaching being “too formal and strict”, while in other contexts teaching was “left to chance” [17, 18].

### Theme 3: Learner Unpreparedness and Engagement Challenges

Students' readiness and engagement were compromised by inadequate exposure, confidence deficits, and contextual factors including the COVID-19 pandemic.

#### Subtheme 3.1: Insufficient Exposure and Confidence Deficits

“Students report inadequate exposure” to bedside teaching was a dominant finding. This created a reinforcing cycle where “low learner confidence limits bedside skill development”. Students described being “underprepared for end-of-life care” and reported “lack of confidence with dying patients”. Fear of making errors manifested as “anxiety about making mistakes in front of peers” and “fear of causing patient harm”. Consequently, “clinical environments [were] perceived as too intense”, contributing to “insufficient practical exposure” [1, 13, 16, 17, 19].

#### Subtheme 3.2: Professionalism and Disengagement

Studies identified “student professionalism challenges” and “student disengagement”. Students were sometimes “unaware of educator workload”, creating interpersonal tension. Passive observation led to “reduced attention and engagement when not directly involved”. Educators noted “limited information about student background”, preventing individualised teaching approaches [16, 17, 19, 24].

#### Subtheme 3.3: Impact of Remote Consultation Modalities

The shift toward remote care, accelerated by COVID-19, created additional barriers. Studies described “loss of peer support and interaction” due to social distancing requirements. The “remote consultation format limits depth of reflection and learning”, while “reduced face-to-face patient-doctor interaction” impaired development of relational clinical skills [9, 16, 24].

### Theme 4: Patient-Related Ethical and Practical Barriers

The patient interface presented ethical and practical barriers to bedside teaching that were distinct from institutional or educator factors.

#### Subtheme 4.1: The Burden on the Patient

There was a “perceived burden on patients” from participation in teaching. “Patient reluctance to participate” was reported as a significant barrier, often related to “patient discomfort during teaching” [1, 12, 16, 19].

#### Subtheme 4.2: Infection Risk Concerns

Infection control considerations introduced novel barriers. Studies cited “concern about COVID transmission” and “persistent nosocomial infection risk”. “High risk groups limit teaching” opportunities, and patients expressed “inability to decline participation” despite infection-related anxieties [12].

#### Subtheme 4.3: Physical Environment and Privacy

Physical constraints at the bedside compounded patient-related barriers. “Lack of privacy in teaching” led to “student embarrassment”. Combined with “discomfort with physical proximity/intimate examinations”, the bedside environment often inhibited effective learning [1, 12].

## Discussion

**Fig. 2:**
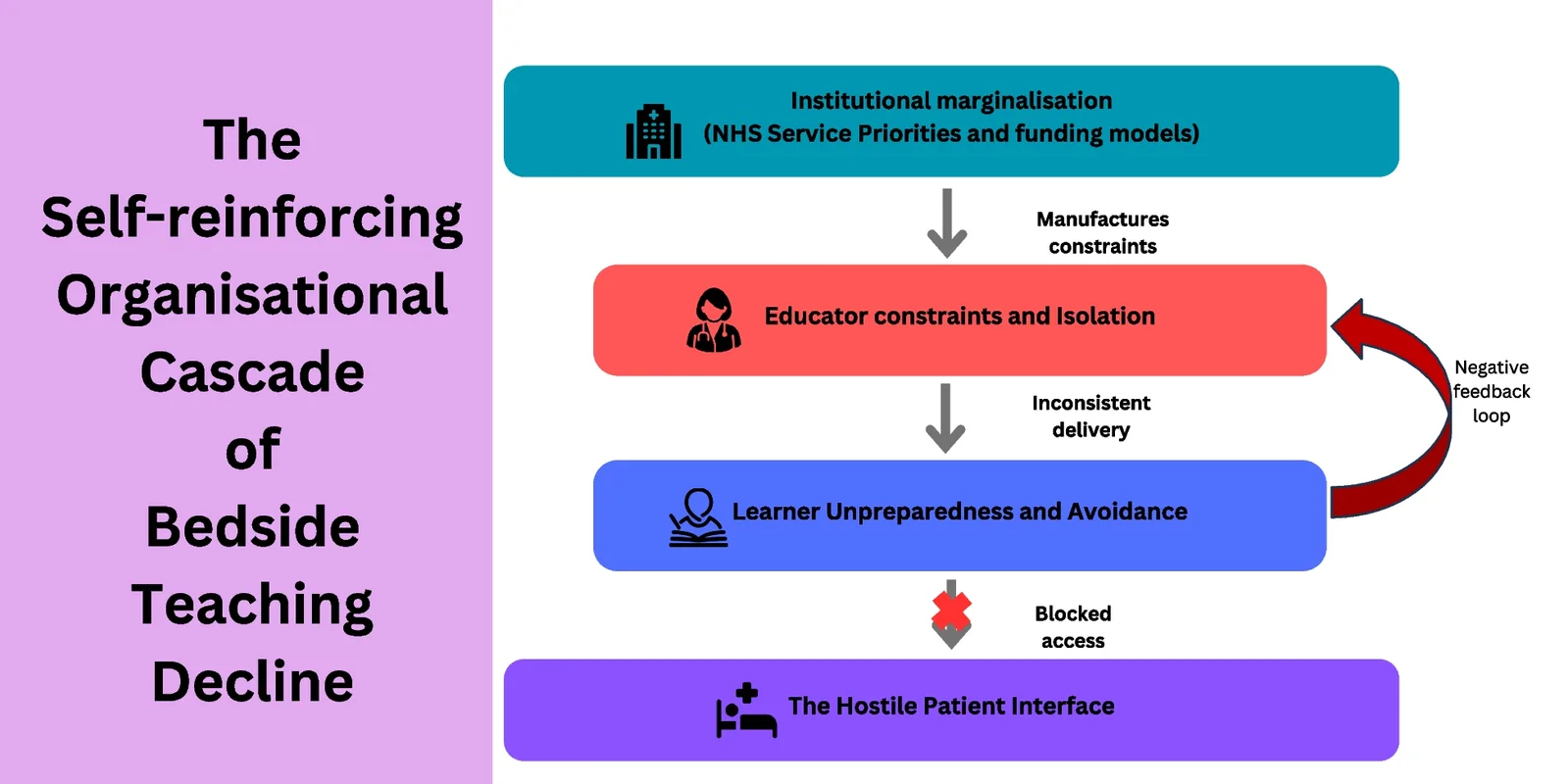
Self-reinforcing organisational cascade constraining bedside teaching in UK clinical education

This systematic review reiterates the traditional catalogue of barriers to bedside teaching, including time constraints, lack of training, and student anxiety, but it also uncovers a more fundamental dysfunction. Our findings suggest that the decline of bedside teaching in the UK is not the result of isolated local impediments but reflects a self-reinforcing organisational cascade. In a healthcare system increasingly oriented toward efficiency and throughput, educational activities are not merely “squeezed”; they are structurally deprioritised. This misalignment between NHS operational priorities and educational mandates creates a cycle where institutional under-resourcing forces educators to triage teaching out of their day, producing unprepared learners whose consequent passivity further discourages educators from investing time (Fig. 2).

The included studies point to structural constraints within teaching environments. In the broader policy context, the senior educator pool is shrinking through early retirement and reduced clinical hours, while medical student numbers are expanding substantially, with plans to nearly double places to 15,000 by 2031 to address projected workforce shortages [5, 20]. This creates a worsening educator-to-learner ratio that intensifies competition for clinical teaching opportunities and places unsustainable strain on an already depleted teaching capacity. The tension becomes particularly acute in the context of workforce expansion. Consistent with this context, a national survey of medical education directors identifies inadequate educator capacity and facilities as barriers to expanding training numbers, emphasising the need for increased funding and infrastructure at an organisational level [21].

The Higher Education Policy Institute (HEPI) report's call for rethinking traditional placements for healthcare students reflects recognition of these capacity constraints [22]. However, we argue that such rethinking must be accompanied by consolidated investment in traditional bedside placements. As medical education increasingly incorporates AI-driven tools, question bank learning, logbook documentation, and screen-based instruction, the irreplaceable hard skills developed at the bedside, namely physical examination technique, procedural competence, and the ability to build rapport and communicate with patients face-to-face, risk becoming casualties of disinvestment. The challenge is not to abandon traditional clinical teaching but to resource it adequately within a training landscape that is expanding in student numbers without a corresponding increase in teaching capacity.

### Differences across clinical settings

Although the four themes described above were evident across both hospital and ambulatory settings, the included studies suggest that the practical expression of these barriers differs by context. In primary care, where GP partners are frequently self-employed and clinical activity is directly linked to practice income, barriers to bedside teaching were often described in terms of funding and backfill, for example “limited resources and funding for teaching” and the absence of “job plan allocation” for staff involved in teaching [9–11, 13]. Primary care-based studies also highlighted the distinct challenge of adapting teaching to remote consultation formats, an issue that is largely specific to general practice service delivery [16]. In hospital settings, barriers centred more on physical infrastructure and service throughput, including a “lack of physical space for student teaching” on wards optimised for bed capacity, and the difficulty of releasing staff from acute clinical duties such as the acute medical take [11, 17].

These differences suggest that, while the underlying organisational cascade we describe operates across settings, interventions may need to account for context. Funding and job-planning reform may be particularly relevant in general practice, whereas protected time and dedicated physical space may be more immediate priorities in hospital-based teaching. These setting-specific differences should be interpreted cautiously, as the included studies were heterogeneous and most were not designed to directly compare inpatient, outpatient and primary care settings. Further primary research directly comparing barriers across these settings would be valuable.

### The self-reinforcing organisational cascade

The central contribution of this synthesis is the identification of a causal feedback loop (Fig. 2) that explains why bedside teaching remains resistant to improvement. The “Institutional Marginalisation” of education (Theme 1) acts as the prime mover. When institutions deprioritise teaching through “lack of job plan allocation” and “limited resources”, they effectively manufacture “Educator Constraints” (Theme 2). Clinicians, struggling to “balance teaching with clinical practice”, inevitably provide variable and opportunistic instruction.

This inconsistent delivery directly produces “Learner Unpreparedness” (Theme 3). Students who experience teaching as “left to chance” fail to develop confidence, leading to “Insufficient Exposure and Confidence Deficits”. Crucially, their subsequent “disengagement” and “professionalism challenges” act as a negative feedback loop, further demoralising educators. Finally, this dysfunction crashes against the “Patient Interface” (Theme 4), where “infection risk” and “lack of privacy” seal off the bedside as a locus of learning.

### The limits of individually targeted interventions

This systemic framing helps explain why interventions targeting individual behaviours have struggled to reverse the decline. Despite decades of faculty development initiatives, teaching awards, and efforts to preserve protected teaching time, bedside teaching has continued its steady decline from 75% of teaching in the 1960 s to less than 20% today [2]. For example, teaching awards attempt to incentivise educators who, our data suggests, are already highly motivated but practically obstructed. Similarly, “protected time” is often illusory in a system where service demands can unilaterally override educational commitments. By treating bedside teaching primarily as a matter of individual skill or will, the medical education community may have obscured a fundamental tension between high-quality clinical teaching and the current design of the NHS service model.

### The hidden curriculum: efficiency as the only value

A further implication of this cascade is the transmission of a “hidden curriculum”. The organisational reality of the NHS can teach learners that, despite rhetorical support for education, service delivery is the metric that most consistently counts. When students observe educators being asked to “work beyond contracted hours” or set aside teaching for waiting list management, they may internalise a professional identity in which the educational role of the physician is marginal [23].

Furthermore, this environment may erode essential generalist and relational skills that depend on repeated, supervised patient contact. Across the included studies, students and educators described insufficient practical exposure, low learner confidence, difficulty engaging with end-of-life care, and variable approaches to clinical skills teaching [14, 19]. These findings suggest that bedside teaching is particularly vulnerable when clinical environments are organised primarily around patient flow, service efficiency and rapid task completion, rather than education.

### Implications for policy and practice

Restoring bedside teaching will require more than isolated educational interventions; it demands a system-wide reorganisation of the NHS that explicitly prioritises teaching within its operational design. Our findings, consistent with recent recommendations from the national survey of medical educators [21], indicate that meaningful change depends on structural redesign, adequate resourcing, and a recalibration of institutional priorities.

This includes honest job planning that recognises teaching cannot be absorbed into existing service pressures without protected time [15], investment in clinical environments that provide the physical space necessary for confidential briefing and debriefing, and formal recognition of the educator role so that teaching carries tangible weight in progression and remuneration [9–11, 17].

At the same time, structural reform must include sustained support for the educators expected to deliver bedside teaching. Our findings point to a need for investment in the educator, particularly as junior clinicians and GP trainees described significant “teacher anxiety about teaching” linked to “limited teacher training” (Subtheme 2.1) [8, 9, 12, 17]. Accessible faculty development programmes, practical bedside teaching skills training, mentorship for newer clinical teachers, and ongoing peer or senior support should therefore be embedded within wider organisational reform. These measures should not be viewed as substitutes for protected time, resourcing and recognition, but as necessary supports that allow clinicians to teach confidently once the system has made space for teaching.

Taken together, these shifts are not local fixes, but components of a broader organisational reform needed to restore bedside teaching as a core function of clinical practice rather than an optional extra squeezed out by service demands.

### Strengths and limitations

This review has several strengths. Adherence to PRISMA 2020 guidelines and PROSPERO registration ensured methodological transparency, while a comprehensive search strategy supplemented by reverse-citation tracking enabled a robust evidence base. By extending the definition of bedside teaching to include primary care encounters, the review reflects the current configuration of UK medical education, where a growing proportion of clinical learning occurs outside hospital wards. The synthesis draws on perspectives from students, junior doctors, consultants, and allied health professionals, offering a broad view of how structural pressures shape the teaching environment across hierarchy levels. The author team, trained outside the UK but now practising within the NHS, brings an outsider-insider lens that supports critical engagement with cultural norms around teaching and allows longstanding practices to be examined with appropriate distance.

Several limitations must also be acknowledged. Restricting the search to peer-reviewed literature may have excluded relevant grey literature, including policy documents and unpublished evaluations, potentially narrowing the range of system-level insights. The included studies were heterogeneous and predominantly small, single-site qualitative projects, limiting the breadth of empirical coverage. Finally, despite structured reflexive processes, the interpretation of the data is inevitably shaped by the authors' own clinical and educational backgrounds, which may influence the framing of structural constraints within the NHS.

## Conclusion

The decline of bedside teaching reflects, above all, structural conditions within the healthcare system that fail to protect space for education. Modern healthcare environments, optimised for efficiency and throughput, can disadvantage the time-intensive and unpredictable nature of clinical instruction. This creates a self-reinforcing cycle in which service pressures sideline educators, students receive inconsistent bedside exposure, and essential “slow” skills, such as physical examination and end-of-life care, become harder to develop and sustain. At the same time, our findings point to a pedagogical dimension that structural reform alone will not resolve: the anxiety and limited preparation reported by newer clinical educators suggest that educator development is a necessary companion to structural change, not a substitute for it. Ultimately, the organisation risks transmitting a hidden curriculum in which, despite rhetorical support for education, service delivery is the metric that most consistently counts.

Sustainable change requires structural reform alongside continued investment in educators. Sustaining and scaling high-quality bedside teaching has become increasingly difficult within the current NHS service model, particularly as student numbers rise without a proportionate expansion of teaching capacity, reflecting the difficulty of delivering teaching at scale rather than a wholesale failure of existing interventions. Restoring clinical education requires system-wide reform that places teaching within the operational design of clinical services. The formation of future doctors should no longer be treated as an optional add-on absorbed into existing workloads, but as a core operational responsibility, supported by educators who are trained, mentored, and resourced to deliver it.

## Supplementary Information

Supplementary Material 1.

## Data Availability

All data generated or analysed during this study are included in this published article and its supplementary information files.
