# Supplementary material for: A systematic review of the current challenges of bedside teaching for medical students in the UK
Source: BMC Med Educ. 2026 Jul 15;26:1608. doi: 10.1186/s12909-026-09931-z (PMC13651361; doi:10.1186/s12909-026-09931-z)
Supplement: Supplementary file 1 [file 12909_2026_9931_MOESM1_ESM.docx]

Supplementary file:

Table of Contents

[Table S1: Search Strategy	2](#_Toc218532088)

[Table S2: CASP Quality Assessment (1)	3](#_Toc218532089)

[Table S3: Reason for exclusion table	4](#_Toc218532090)

[Reference:	6](#_Toc218532091)

Table S1. MEDLINE search strategy

| **Search Step** | **Search Terms** | **Results** |
| --- | --- | --- |
| **S1** | MH "Teaching+") OR “bedside teaching” OR (MH "Teaching Rounds") | 100,305 |
| **S2** | XB (“bedside teaching” OR “clinical teaching” OR “ward-based teaching” OR “patient-centered teaching” OR “bedside instruction” OR “experiential learning” OR “case-based learning”) | 7,940 |
| **S3** | S1 OR S2 | 104,706 |
| **S4** | XB (“challenges” OR “barriers” OR “difficulties” OR “limitations” OR “obstacles” OR “constraints” OR “problems”) | 1,997,871 |
| **S5** | (MH "Students, Medical") OR "medical students" | 182,155 |
| **S6** | XB (“medical students” OR “student doctors” OR “medical trainees” OR “clinical learners” OR “undergraduate medical education”) | 57,925 |
| **S7** | S5 OR S6 | 184,155 |
| **S8** | XB (“United Kingdom” OR “UK” OR “NHS” OR “Britain” OR “England” OR “Scotland” OR “Wales” OR “Northern Ireland”) | 330,199 |
| **S9** | S3 AND S4 AND S7 AND S8 | 159 |
| **S10** | S3 AND S4 AND S7 AND S8 AND 2014-2025 | 71 |

# Table S2. CASP qualitative appraisal of included studies (1).

| Author/Year | Q1 | Q2 | Q3 | Q4 | Q5 | Q6 | Q7 | Q8 | Q9 | Q10 |
| --- | --- | --- | --- | --- | --- | --- | --- | --- | --- | --- |
| Gadoud et al 2018 | Yes | Yes | Yes | Yes | Yes | Yes | Yes | Yes | Yes | Yes |
| Zamoyski and Alberti 2018 | Yes | Yes | Yes | Yes | Yes | Yes | Yes | Yes | Yes | Yes |
| Harrison et al 2019 | Yes | Yes | Yes | Yes | Yes | Yes | Yes | Yes | Yes | Yes |
| Harris et al 2024 | Yes | Yes | Yes | Yes | Yes | Can't tell | Yes | Yes | Yes | Yes |
| Barber et al 2019 | Yes | Yes | Yes | Yes | Yes | Yes | Yes | Yes | Yes | Yes |
| Jones et al 2017 | Yes | Yes | Yes | Yes | Yes | Yes | Yes | Yes | Yes | Yes |
| Gray et al 2016 | Yes | Can't tell | Yes | Yes | Yes | Can't tell | Yes | Can't tell | Yes | Yes |
| Shone et al 2025 | Yes | Yes | Yes | Yes | Yes | Can't tell | Yes | Yes | Yes | Yes |
| Harding et al 2015 | Yes | Can't tell | Yes | Yes | Yes | Can't tell | Yes | Yes | Yes | Yes |
| Blake et al 2014 | Can't tell | Yes | Yes | Yes | Yes | Can't tell | Yes | Yes | Yes | Yes |
| Jones and Rai 2015 | Yes | Can't tell | Yes | Yes | Yes | Can't tell | Yes | Yes | Yes | Yes |
| Chew and O'Dwyer 2020 | Yes | Yes | Yes | Yes | Yes | Can't tell | Can't tell | Yes | Yes | Yes |
| Al-bedaery et al 2022 | Yes | Yes | Yes | Yes | Yes | Can't tell | Yes | Yes | Yes | Yes |
| Hayden et al 2021 | Yes | Yes | Yes | Yes | Yes | Can't tell | Yes | Yes | Yes | Yes |

Key:

Q1. Was there a clear statement of the aims of the research?

Q2. Is a qualitative methodology appropriate?

Q3. Was the research design appropriate to address the aims of the research?

Q4. Was the recruitment strategy appropriate to the aims of the research?

Q5. Was the data collected in a way that addressed the research issue?

Q6. Has the relationship between researcher and participants been adequately

Q7. Have ethical issues been taken into consideration?

Q8. Was the data analysis sufficiently rigorous?

Q9. Is there a clear statement of findings?

Q10. How valuable is the research?

Table S3. Reasons for exclusion of full‑text articles assessed for eligibility.

| No. | Study title | Author | Year | Reason for Exclusion |
| --- | --- | --- | --- | --- |
| 1 | Bedside teaching: an invaluable tool in undergraduate medical education | Ruddock | 2021 | Conference poster/abstract; not a peer-reviewed qualitative study. |
| 2 | Bedside teaching: everybody's but nobody's responsibility | Gimson et al | 2019 | Not qualitative research; commentary on improvement strategies. |
| 3 | How we established a new undergraduate firm on a Medical Admissions Unit | Nazir et al | 2014 | Not qualitative research; descriptive implementation overview, not barriers-focused. |
| 4 | Enhancing medical students engagement in Obstetrics and Gynaecology (O&G) during Covid-19 | Siriboe et al | 2021 | Conference poster/abstract; not a peer-reviewed qualitative study. |
| 5 | Undergraduate teaching in geriatric medicine in the University Hospital of Wales | Pathy | 1974 | Outside timeframe (2014-2025); descriptive programme report, not qualitative barriers research. |
| 6 | Bridging the education-action gap: a near-peer case-based undergraduate ethics teaching programme | Kong and Knight | 2017 | Not about bedside-teaching barriers; focuses on an ethics teaching programme. |
| 7 | Factors affecting consultant attitudes to undertaking undergraduate medical student teaching in the UK: a systematic review | Isobel et al | 2021 | Systematic review; not primary qualitative research on bedside-teaching barriers. |
| 8 | Community-based teaching: The challenges. | Murray and Modell | 1999 | Outside timeframe (2014-2025); discussion/commentary, not empirical qualitative research on bedside teaching. |
| 9 | How does dyslexia impact on the educational experiences of healthcare students? A qualitative study | Ali et al | 2019 | Not about bedside-teaching barriers; focuses on dyslexia and educational experiences. |
| 10 | Hostile teaching hospitals and friendly district general hospitals: final year students’ views on clinical attachment locations | Parry et al | 2002 | Outside timeframe (2014-2025); quantitative survey, not qualitative barriers research. |
| 11 | Medical student experiences of gynaecological teaching aids kings college london UK | Wilson and Bruce | 2018 | E-poster/compilation; not a qualitative study and unrelated to bedside-teaching barriers. |
| 12 | Robotic simulation experience in undergraduate medical education: a perspective | Naik and Mandal | 2020 | Not about bedside-teaching barriers; simulation-focused education study. |
| 13 | Running a workshop on clinical bedside teaching: a practical guide. | Bullimore | 1993 | Outside timeframe (2014-2025); not about bedside-teaching barriers. |
| 14 | Service Increment for Teaching (SIFT): A review of its origins, development and current role in supporting undergraduate medical education in England and Wales. | Clack et al | 1999 | Outside timeframe (2014-2025); policy/finance review, not qualitative barriers research. |
| 15 | Stroke medicine teaching in UK medical schools: A design for a nationwide undergraduate stroke education module | McGurgan et al | 2019 | Unrelated to bedside teaching; tool validation study, not qualitative barriers research. |
| 16 | Teach paediatrics and inspire: using QIP methodology in education promotes prospective trainees’ interest in a career in paediatrics | Rosen et al | 2021 | Conference poster/QI project; not qualitative barriers research. |
| 17 | Training tomorrow’s doctors in the pre-op clinic | Cousland et al | 2013 | Outside timeframe (2014-2025); evaluation/audit study, not qualitative barriers research. |
| 18 | Ultrasound skills teaching in UK medical education: A systematic review | McCormick et al | 2023 | Systematic review on ultrasound teaching; not primary qualitative barriers research. |
| 19 | Developing junior doctor-delivered teaching | Qureshi et al | 2013 | Programme description/evaluation; not qualitative barriers research. |
| 20 | GP trainees as teachers: a rapid review of the barriers, facilitators and outcomes | Bull et al | 2024 | Rapid review (secondary research) focused on GP trainee teaching rather than primary qualitative evidence on barriers to bedside teaching in UK clinical settings. |

# Reference:

1. Critical Appraisal Skills Programme (2023). CASP Qualitative Studies Checklist. [online] Available at: https://casp-uk.net/casp-tools-checklists/qualitative-studies-checklist/. Accessed: 05 January 2025
